# Vitiligo-like lesions induced by cyclin-dependent kinase 4/6 inhibitor Palbociclib: a case report and literature review

**DOI:** 10.3389/pore.2023.1611115

**Published:** 2023-07-06

**Authors:** Shan Gao, Guanjing Wei, Yanrong Hao

**Affiliations:** ^1^ Medical Oncology Division 1, Clinical Oncology Center, People’s Hospital of Guangxi Zhuang Autonomous Region, Nanning, China; ^2^ Department of Dermatology and Venereology, People’s Hospital of Guangxi Zhuang Autonomous Region, Nanning, China

**Keywords:** breast cancer, CDK4/6 inhibitors, Palbociclib, adverse events, vitiligo-like lesions

## Abstract

Endocrine therapy has played an essential role in hormone receptor-positive (HR+), human epidermal growth factor receptor 2-negative (HER2−) breast cancer. With the continuous development of endocrine targeting drugs, especially the emergence of selective cyclin-dependent kinase (CDK4/6) inhibitors, the overall survival time in patients with HR+HER2− advanced breast cancer has been greatly improved. Their adverse reactions also need more attention in response to the climbing number of CDK4/6 inhibitors. The common side effects of CDK4/6 inhibitors were hematological toxicity, diarrhea, and liver function damage. Skin toxicity related to CDK4/6 inhibitors was rare. We describe herein our preliminary observation of one HR+HER2− advanced metastatic breast cancer patient diagnosed with vitiligo-like lesions after 10 months of taking Palbociclib. Hoping to share our experience to increase the clinician awareness of this unusual adverse and contribute to the information in the literature.

## Background

Breast cancer is women’s most common malignant tumor globally [1]. Endocrine therapy is a reasonably necessary treatment for HR+ breast cancer. In recent years, with the emergence and widespread application of endocrine-targeted drugs, treatment strategies have resulted in revolutionary changes, further improving patients’ overall survival time and quality of life. CDK4/6 inhibitors are one of the most representative stars. Combination with aromatase inhibitors (AIs) or Fulvestrant has become the preferred first-line treatment for HR+HER2- advanced breast cancer patients [2, 3]. Currently, three CDK4/6 inhibitors worldwide used in the world: Ribociclib, Palbociclib, and Abemaciclib [4, 5]. The most common adverse events (AEs) consist of neutropenia, leukopenia, anemia, fatigue, liver dysfunction, and diarrhea. What’s more, treatment with CDK 4/6 inhibitors also be associated with several potential dermatologic toxicities, including alopecia, which is the most frequent [6]. Vitiligo-like lesions induced by CDK4/6 inhibitors are rare, although some case reports have described vitiligo-like lesions caused by Ribociclib [7, 8].

Here we describe a rare case of vitiligo-like lesions developing after treatment with Palbociclib, a CDK4/6 inhibitor. This 58-year-old woman did not experience any discomfort and insisted on taking the drug for further use of the regimen. We adopted the “watch and wait” strategy, and her progression-free survival time has exceeded 24 months. Intending to share our experience to raise awareness among clinicians and contribute to the literature, we present the following information based on the CARE reporting checklist [9].

## Case presentation

### Ethics

Written informed consent was obtained from the participant for the publication of any potentially identifiable images or data included in this article.

A 58-year-old female patient was diagnosed with right-side breast invasive lobular carcinomas through imaging examinations and pathological assessment in November. 2020. Ultrasonography revealed a 9*8 mm mass in the seven o’clock direction, with BI-RADS-Us stage IVb. Immunohistochemistry showed that the tumor cells were estrogen receptor/progesterone receptor-positive, and HER2-, Ki-67 5%–10% positive, the clinical stage is cT1N0M1, stage IV, the molecular subtype is Luminal A, with multiple bone metastases at the same time.

As she was in a premenopausal state at that time, according to the guidelines, she started Palbociclib and letrozole as the first-line therapy after receiving ovarian ablation in January 2021.

**FIGURE 1:**
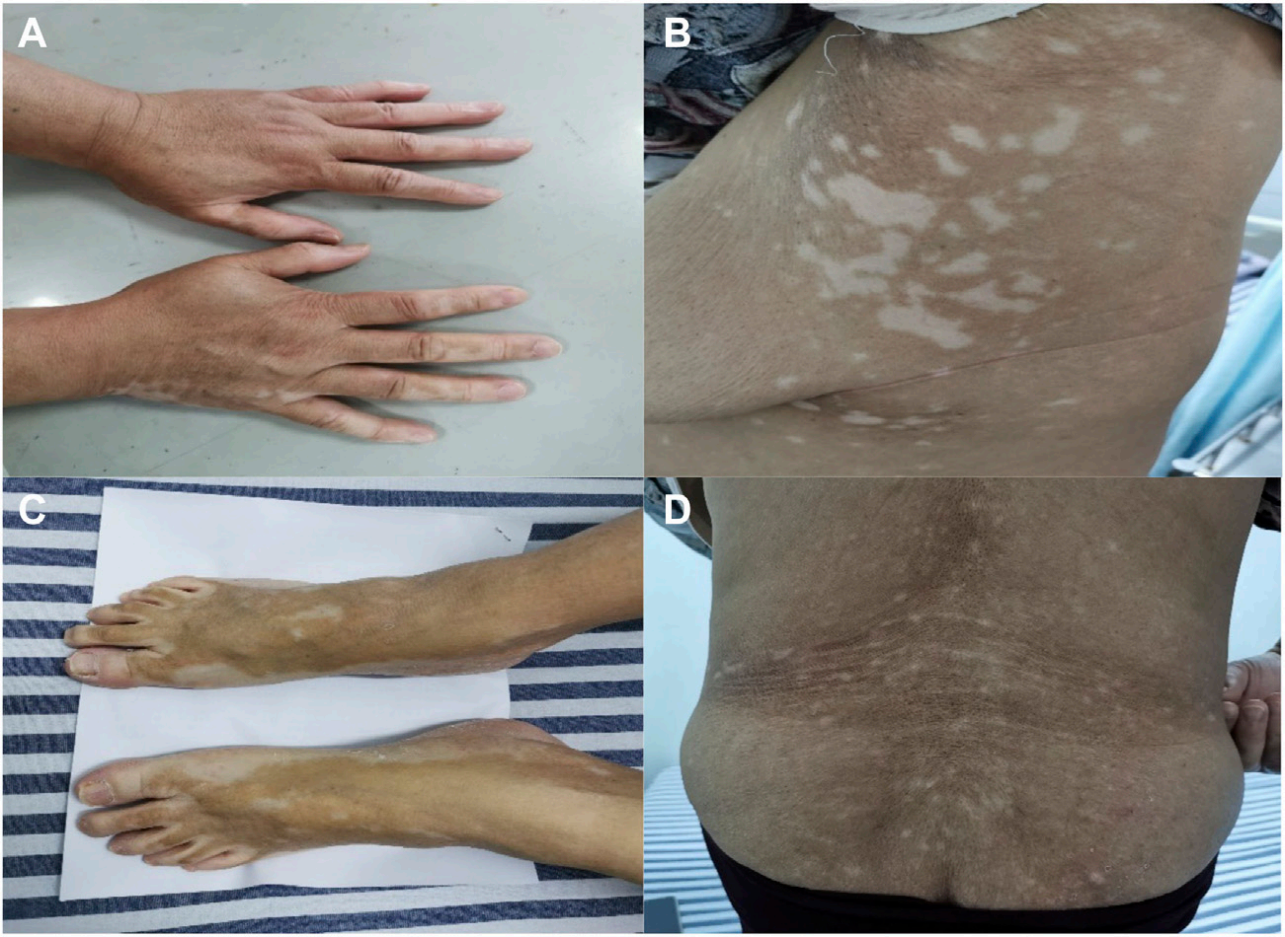
Clinical images. The non-segmental/bilateral depigmented macules over the hands **(A)**, fossa axillaries **(B)**, and feet **(C)**. Vitiligo-like lesions over the lower back **(D)**. Multiple irregularly, well-demarcated, hypopigmented lesions surrounded by normal skin.

**FIGURE 2:**
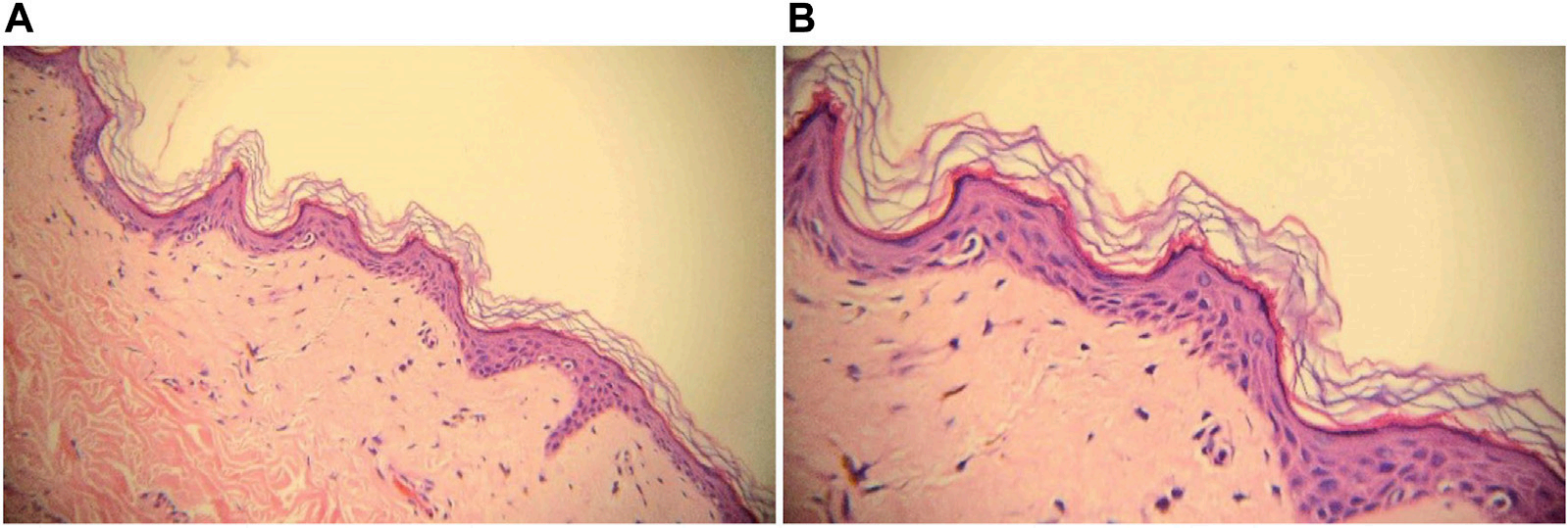
Biopsy with H&E staining of depigmented macule, ×20 magnification **(A)** and ×40 magnification **(B)**. Melanocytes and melanin granules in the epidermis of the leukoplakia area were absent.

Ten months later, the patient presented with multiple irregularly, well-demarcated, hypopigmented lesions over both axillary fossa that gradually spread to the arms, legs and lower back without pruritus or erythema. She did not have any other comorbidities, nor did she have a family history of vitiligo, hyperthyroidism, or systemic lupus erythematosus. A dermatological examination revealed multiple depigmented macules of varying sizes over the hands, forearms, thighs, feet, fossa axillaries, and lower back (Figure 1). A wood lamp examination showed blue-white patches with clear boundaries and increased pigmentation at the edges of white patches. Skin biopsies were taken from depigmented macules; the histological pathology shows the complete absence of melanocytes and melanin, in addition to a mild infiltration of inflammatory cells around the blood vessels (Figure 2). Based on these examination results, non-segmental vitiligo of grade 2 was diagnosed, covering approximately 6% of the body surface area (BSA). Since she did not experience any discomfort, she refused treatment with medicine or laser therapy. She continues to take Palbociclib and letrozole to date.

As of January 2023, the tumor and vitiligo are stable, and the progression-free survival time has exceeded 24 months.

## Discussion

Paloma-2 [10], a randomized controlled double-blind phase III clinical study investigating the efficacy and safety of Palbociclib in combination with letrozole in patients with primary HR+HER2− advanced breast cancer. A total of 666 patients were enrolled in the study. Median progression-free survival was 24.8 months for Palbociclib -letrozole versus 14.5 months for placebo-letrozole (hazard ratio, 0.58; 95% CI, 0.46 to 0.72; *p* < 0.001). The most common treatment-related adverse events (AEs) of grade 3 or 4 were neutropenia, leukopenia, anemia, and fatigue. Dermatologic AEs reported in this article included alopecia (Any grade 32.9%), rash (Any grade 17.8, Grade 3 0.9%), and dry skin (Any grade 32.9%). No dermatologic AEs of vitiligo-like lesions were reported.

Vitiligo is an acquired pigmentary autoimmune disorder consisting of developing hypopigmented macules due to the selective loss of melanocytes. Possible mechanisms for the development of vitiligo include autoimmunity, oxidative stress, melanocyte self-destruction, and genetic predisposition [7, 11]. It’s not life-threatening. Nevertheless, it affects patients’ psychosocial health, interpersonal communication, and health-related quality of life [12]. In patients treated with immune checkpoint inhibitors, vitiligo-like lesions caused by antineoplastic drugs are occasionally reported. We were also surprised to find occasional reports of vitiligo-like lesions associated with B-cell leukemia/lymphoma-2 (Bcl-2) inhibitor Venetoclax [13].

Vitiligo-like lesions induced by cyclin-dependent kinase 4/6 (CDK4/6) inhibitors are rare, and the pathogenesis of vitiligo-like lesions induced by CDK4/6 inhibitors is also poorly understood. Zhou [14] hypothesized that CDK inhibitors can block the cell cycle and deregulate the expression of small ubiquitin-like modulators expression in keratinocytes, eventually resulting in the occurrence of vitiligo.

To our knowledge, only three cases of Pabociclib-induced vitiligo-like lesions have been reported in the literature. The data on the previous reports were summarized data are enumerated in Table 1. Sollena [15], on behalf of the European Network for Cutaneous ADverse event to Oncologic drugs (ENCADO) group, first reported two cases of vitiligo-like lesions after using Palbociclib in the treatment of 16 patients with metastatic breast cancer in six European university-based centers. Both two patients experienced cutaneous pruritus. Patient No. 1 was affected with vitiligo-like lesions in 60% of BSA after taking 8 months of Palbociclib and Fulvestrant was treated with oral corticosteroids and calcineurin inhibitors, the outcome of dermatologic treatment is PR, the lesions reached an improvement and the area reduced. Patient No. 2 found vitiligo-like lesions in 12 months of using Palbociclib and Letrozole was treated with topical oral corticosteroids. No. 3 is a patient who appeared hypopigmented lesions after 1 month of changing from Ribociclib to Pabociclib. At the same time, she decided to refuse to take any treatment, and the cutaneous lesions were stable within a 4-month follow-up [16].

**TABLE 1:** | Palbociclib-induced vitiligo-like lesions in the literature.

Patient’s No.	Authors	Age at vitiligo onset	Type of treatment	Hormone therapy associated	Clinical response	Vitiligo-like lesion body sites	% of BSA^†^ with vitiligo-like lesions	No. of cycle at diagnosis	Treatment of vitiligo-like lesions	Outcome of dermatologic treatment
1	Sollena et al. (2021) [15]	42	Palbociclib 125 mg	Fulvestrant 500 mg	SD^‡^	arms, thighs, legs, forearms, face, dorsal aspects of the hands	60	8	Calcineurin inhibitors + Oral corticosteroids	PR
2	Sollena et al. (2021) [15]	63	Palbociclib 125 mg	Letrozole 2.5 mg	PR^§^	face, arms, chest, back	18	12	Topical corticosteroids	NR
3	Romagnuolo et al. (2023) [16]	47	Palbociclib 125 mg	Letrozole 2.5 mg	NR^¶^	chest, upper arms, and legs	NR	1	None	SD

^†^BSA body surface area, ^‡^SD stable disease, ^§^PR partial response, ^¶^NR no response

Our patient was diagnosed with vitiligo after 10 months of Palbociclib and letrozole treatment. Her occurrence time of vitiligo-like lesions is approximately consistent with the patients’ described in Sollena’s study. As she did not experience any discomfort, such as inflammation or pruritus, she does not agree to any additional medication. Tumors and vitiligo were observed to remain stable for up to now, more than 14 months. We have reason to suspect that these vitiligo-like skin lesions are related to Palbociclib in the absence of prior evidence that letrozole may cause specific hypopigmented macules. We need to be vigilant about this rarely-seen adverse; counselling patients regarding the possibility of vitiligo¬like lesions prior to initiation of Palbociclib may help lessen the impact of this adverse event on the patient’s mental health and quality of life.

## Conclusion

Our case adds to the knowledge gained from the existing case reports in the literature. Firstly, vitiligo-like skin lesions can be encountered late in Palbociclib therapy. Second, the range of vitiligo-like skin lesions does not show remission during an intermission in treatment, which provides evidence that these lesions induced by Palbociclib are irreversible. Future large-scale clinical studies are needed to provide more evidence for this hypothesis.

## Data Availability

The original contributions presented in the study are included in the article/supplementary material, further inquiries can be directed to the corresponding author.
